# Clinical application of upper limb fracture fixator in emergency treatment of upper limb fracture

**DOI:** 10.12669/pjms.39.6.7097

**Published:** 2023

**Authors:** Lianghao Xiong, Jintao Le, Chun Zhao, Rongxia Yuan, Mai Yang, Hai Shen

**Affiliations:** 1 Lianghao Xiong, Department of Emergency, Sichuan Province Orthopedics Hospital, Chengdu 610041, Sichuan, China; 2 Jintao Le, Department of Emergency, Sichuan Province Orthopedics Hospital, Chengdu 610041, Sichuan, China; 3 Chun Zhao, Department of Emergency, Sichuan Province Orthopedics Hospital, Chengdu 610041, Sichuan, China; 4 Rongxia Yuan, Department of Emergency, Sichuan Province Orthopedics Hospital, Chengdu 610041, Sichuan, China; 5 Mai Yang, Department of Emergency, Sichuan Province Orthopedics Hospital, Chengdu 610041, Sichuan, China; 6 Hai Shen, Department of Emergency, Sichuan Province Orthopedics Hospital, Chengdu 610041, Sichuan, China

**Keywords:** Emergency treatment, First aid, Fracture upper limb fixator, Upper limb fracture

## Abstract

**Objective:**

To compare and analyze the curative effect of fracture upper limb fixator and traditional splint in emergency treatment of upper limb fracture.

**Methods:**

This is a prospective study. A total of 80 patients with upper limb fractures admitted to the Emergency Department of Sichuan Province Orthopedic Hospital from December 2021 to August 2022 were prospectively selected as subjects. They were divided into two groups according to the random number table method: Patients in the control group were treated with traditional splint, while those in the observation group were treated with medical adjustable upper limb fixator. The clinical efficacy, treatment time, pain, fitness, probability of secondary injury and complications were compared between the two groups.

**Results:**

After treatment, the excellent and good rate in the observation group (75.00%) was higher than that in the control group(52.50%). There was no statistically significant difference in the treatment time between the two groups. There was no significant difference in the probability of secondary injury between the two group. Statistically significant difference was observed in the comparison of pain conditions between the two groups. The total fitness rate of the observation group (97.50%) was higher than that of the control group (75.00%). The total incidence of complications in the observation group (2.50%) was lower than that in the control group (20.00%), with a statistically significant difference.

**Conclusion:**

Fracture upper limb fixator shows various benefits in first aid of upper limb fractures, such as improving the clinical efficacy of patients with upper limb fractures, ameliorating pain, improving fitness effect, and reducing the probability of complications.

## INTRODUCTION

Fracture is a common clinical symptom, in which the integrity of the specific phalanges is destroyed or the continuity is interrupted. Typically, the bones can be fractured when they are subjected to forces that exceed their maximum strength.^1^,^2^ Most fractures are caused by direct or indirect violence, such as falls, impacts, and traffic injuries, and some patients with cumulative strain and bone diseases also have fractures of varying degrees.^3^,^4^ Upper limb fracture refers to the fracture of the shoulder, upper arm, elbow, forearm, wrist and hand.^5^-^8^ Patients with upper limb fractures are mostly manifested as local pain, swelling, mobility disorder, etc., which have a great impact on the quality of life. Therefore, patients should go to the hospital for treatment as soon as possible after fracture.^9^ Emergency department, as the department with the largest number of diseases and the heaviest rescue and management tasks in the hospital, is one of the most important treatment departments in the hospital. Because of the sudden onset and great pain of fracture, most patients choose to go to the emergency department for treatment.

The diagnosis and treatment of fracture in emergency treatment can assist patients to make comprehensive judgment after admission and lay the foundation for follow-up treatment. The upper limb fracture fixator is a common tool in emergency treatment of upper limb fracture. However, the traditional fixator has some limitations in matching the length of the affected limb, and it is easy to cause pressure injury to patients due to the poor air permeability of the material.^10^ In contrast, adjustable upper limb fixator is a new external fixator for upper limb fracture, featuring convenience, time-saving and safety.^11^ However, few studies have been carried out on the application of adjustable upper limb fixator in emergency treatment of upper limb fracture. In view of this, we recruited 80 patients into the study based on our own clinical experience, in order to investigate the clinical application effect of upper limb fixator in emergency treatment of upper limb fracture.

## METHODS

This is a prospective study. A total of 80 patients with upper limb fractures admitted to the Emergency Department of Sichuan Province Orthopedic Hospital from December 2021 to August 2022 were prospectively selected as subjects. They were divided into two groups according to the random number table method the control group and the observation group, with 40 cases in each group. This study complied with the Declaration of Helsinki.

### Ethical Approval:

This study has been approved by the medical ethics committee of Ethical Approval: Sichuan Province Orthopedics Hospital (NO.:2017-6-30-1; date: June 30, 2017), and written informed consent was obtained from all participants.

The guidelines for the diagnosis of upper limb fracture were drawn up based on “Therapeutics of Upper Limb Fractures with Integrated Traditional Chinese and Western Medicine”^12^: (1) Clinical signs include pain of varying degrees, swelling of the affected area, ecchymosis, and deformities in different situations such as angulation, rotation, and overlapping; (2) Local tenderness, percussion pain, abnormal activity, and bone rubbing sound; (3) X-ray examination showed the existence of continuous and smooth dense lines with low density shadow. (4) CT showed that the continuity of bone and skin was interrupted, and some of them showed multiple fractured pieces, fracture displacement, and swelling images of surrounding soft tissues. (5) Magnetic resonance imaging showed long T1 signal and high signal shadow in T2 fat-suppressed images.^13^

### Inclusion criteria:

- Patients who meet the above diagnostic criteria and are diagnosed with upper limb fracture after seeing a doctor;
- Patients with fractures of humerus, ulna and radius;
- Patients over 18 years of age;
- Patients with clear examination results;
- Patients in emergency department within six hours after fracture;
- Patients who meet the indications of upper limb fixator treatment and are suitable for upper limb fixator treatment;
- Patients who know the research content, know the pros and cons and have signed the informed consent form.

### Exclusion criteria:

- Patients with malignant tumor, coagulation dysfunction, other fractures, abnormal liver and kidney function;
- Patients with fracture history within three months before joining the group;
- Patients who were treated with self-fixation before joining the group;
- Patients with bone diseases such as osteoporosis;
- Patients with poor compliance and obvious uncooperative performance during treatment;
- Patients who do not receive outpatient reexamination after treatment;
- Patients transferred to hospital halfway;
- Pregnant women.

The traditional splint consists of rectangular planks of different types. The external fixator consists of a length control lock, an angle chuck, an alloy bracket and a memory sponge. Patients in the control group were treated with traditional splint, while those in the observation group were treated with medical adjustable upper limb fixator. Control group: In case of an open fracture, first bandage the wound, then cover the wound with dressing. If there is no skin injury, select the appropriate splint directly according to the fracture position of the patient, fix the splint on the dorsal, external and internal sides of the affected limb and the palmar or external, internal, anterior and posterior sides, and fix it with bandages at the two sides and middle positions of the fixed splint with the assistance of a physician assistant, with moderate strength. Too loose or too tight is not advisable. After the swelling of the limbs subsides, adjust the tightness of the splint to ensure the fixation effect. Observation group: In case of an open fracture, the dressing and dressing methods are the same as those of the control group. Rehabilitation training includes finger flexion and extension, wrist flexion and extension, rotation training, shoulder flexion and extension and adduction and abduction training, elbow flexion and extension training, forearm rotation training and so on.

If there is no skin injury, an adjustable memory upper limb fixator is used to fix the fracture directly. After fracture reduction, place the affected limb in a horizontal position, place the fixator under the affected limb, and wrap the arm. Adjust the automatic buckle according to the length and dimension of the affected limb to conform to the physiological curvature of the arm. After setting the functional position, adjust the elbow and wrist adjuster to fix the affected limb. Finally, the affected limb is suspended and fixed on the neck with forearm fixing belt.

### Observation index:

(1) Clinical efficacy. X-ray review was performed one month after treatment. Excellent: fracture displacement distance within 4 mm; Good: fracture displacement distance within 4-10 mm; Medium: fracture displacement distance within 11-20 mm; Poor: fracture displacement distance more than 20 mm. Excellent and good rate = excellent rate + good rate^14^; (2) Treatment time and probability of secondary injury. The overall treatment time of patients and the probability of secondary injury during treatment were recorded; (3) Pain according to the classification criteria of the World Health Organization, pain can be divided into five grades, Grade-0: painless; Grade-I: mild pain without medication; Grade-II: severe pain and need medication control; Grade-III: severe pain, which is persistent pain and cannot be relieved without medication; Grade-IV: severe pain with changes in blood pressure and pulse^15^; (4) Fitness the fitness between the fixator and the body of the two groups was observed by the same physician, which can be divided into excellent fitness, relative fitness and non-fitness, with the total fitness=excellent fitness rate + fitness rate; (5) Complications. The incidence of infection, nerve injury, deformity, and pressure sore within one month after treatment was recorded. There was no local tenderness and longitudinal percussion pain. There was no abnormal activity in the local area. X-ray showed that the fracture line was blurred and continuous callus passed through the fracture line. Lift the one kilogram weight forward for one minute. No deformation was observed continuously for two weeks. Removable splint / external fixator.

### Statistical Analysis:

All data in this study were processed using statistical software SPSS 22.0. The counting data such as clinical efficacy, probability of secondary injury, fitness and complications were represented by (n, %), and *χ^2^* test was performed, while grade data such as pain was tested by rank sum test. Measurement data, such as treatment time, were indicated by (*χ̅*±*S*), and *t* test was performed. P<0.05 indicates a statistically significant difference. Graph Pad Prism eight software was used for drawing.

## RESULTS

In the observation group, there were 14 males and 26 females, ranging in age from 29 to 64 years old, with an average of (46.35 ± 12.18) years old. 10 and 30 cases had fractures of the proximal forearm and distal radius respectively, while 28, five and seven cases suffered fractures due to fall injuries, falls, and others, respectively. In the control group, there were 15 males and 25 females, ranging in age from 29 to 66 years old, with an average of (46.98 ± 11.50) years old; 10 and 30 cases had fractures of the proximal forearm and distal radius respectively, while 28, five and seven cases suffered fractures due to fall injuries, falls, and others, respectively. No statistically significant difference was observed in the baseline data between the two groups (P > 0.05), which was comparable.

**Fig.1:**
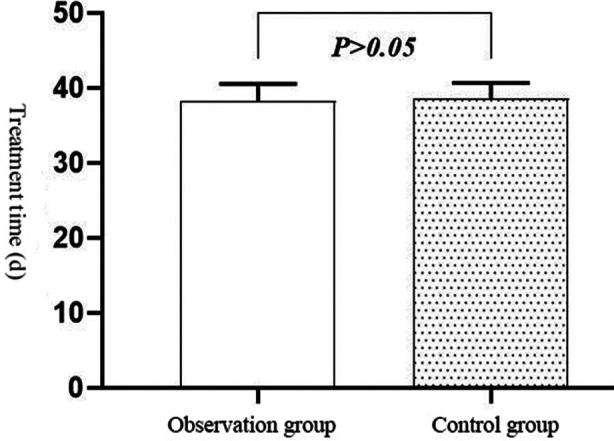
Comparison of treatment time between the two groups.

The excellent and good rate in the observation group (75.00%) was higher than that in the control group (52.50%), with a statistically significant difference (P < 0.05) Table-I. The treatment time of the observation group was (38.33 ± 2.22) d, and that of the control group was (38.60 ± 2.07) d, with no statistically significant difference between the two groups (t = -0.572, P > 0.05). Fig.1: No patients in the observation group suffered secondary injury during the treatment, while one patient in the control group suffered such injury during the treatment, with an incidence rate of 2.50%. There was no significant difference in the probability of secondary injury between the two groups (*χ^2^=*0.000, P>0.05).

**Table-I:** Comparison of clinical efficacy between the two groups [*n* (%)].

Group	n	Excellent	Good	Medium	Poor	Excellent and good rate
Observation group	40	10 (25.00)	20 (50.00)	8 (20.00)	2 (5.00)	30 (75.00)
Control group	40	7 (17.50)	14 (35.00)	15 (37.50)	4 (10.00)	21 (52.50)
*χ^2^*						4.381
*P*						0.036

In the observation group, the proportion of pain Grade-0 was the highest, while that of pain Grade-IV was the lowest. In contrast, in the control group, the proportion of pain Grade-I was the highest, while that of pain Grade-IV was the lowest. No statistically significant difference was observed in the comparison of pain conditions between the two groups (P < 0.05) Table-II.

**Table-II:** Comparison of pain between two groups [*n* (%)].

Group	n	Grade 0	Grade I	Grade II	Grade III	Grade IV
Observation group	40	25 (62.50)	8 (20.00)	4 (10.00)	2 (5.00)	1 (2.50)
Control group	40	14 (35.00)	16 (40.00)	5 (12.50)	3 (7.50)	2 (5.00)
*Z*		-2.153				
*P*		0.031				

A total of 80 patients were included, and 44 patients had excellent fitness between the fixture and the body, with a probability of 55.00% (44/80). The total fitness rate in the observation group (97.50%) was higher than that in the control group (75.00%), with a statistically significant difference (P < 0.05) Table-III.

**Table-III:** Comparison of fitness between the two groups [*n* (%)].

Group	n	Excellent fitness	Relative fitness	Non-fitness	Total fitness rate
Observation group	40	25 (62.50)	14 (35.00)	1 (2.50)	39 (97.50)
Control group	40	19 (47.50)	11 (27.50)	10 (25.00)	30 (75.00)
*χ^2^*	-				8.538
*P*	-				0.003

A total of 80 patients were included, and nine patients developed complications within one month after treatment, with a complication rate of 11.25% (9/80). The total complication rate of the observation group (2.50%) was lower than that of the control group (20.00%), with a statistically significant difference (P < 0.05) Table-IV.

**Table-IV:** Comparison of complications between the two groups [*n* (%)].

Group	n	Infection	Nerve injury	Deformity	Pressure sore	Total incidence
Observation group	40	1 (2.50)	0 (0.00)	0 (0.00)	0 (0.00)	1 (2.50)
Control group	40	4 (10.00)	0 (0.00)	0 (0.00)	4 (10.00)	8 (20.00)
*χ^2^*	-					4.507
*P*	-					0.034

## DISCUSSION

In this study, 40 patients with upper limb fracture were treated with adjustable upper limb fixator, only one patient developed skin infection within one month of treatment, and the total complication rate was 2.50%, which was significantly lower than that of 20.00% in the control group. This shows that the materials and structures of adjustable upper limb fixator can really protect skin. The reason is that the inner layer of the fixator used in this study is memory sponge, which is soft and porous. While increasing patient comfort, it prevents pressure injuries and is suitable for long-term contact with the skin.^16^,^17^

The emergency department is the epitome of the overall work of the hospital, which can directly reflect the quality of emergency medical care and nursing work in the hospital. It occupies a substantial position in the modern emergency medical system. The emergency department of orthopedics mainly treats acute attacks of osteoarthrosis or diseases with severe pain, such as fracture and dislocation of osteoarthrosis.^18^-^20^ With the rapid industrialization process in China and the accelerated pace of people’s life, car crashes and accidents occur frequently, resulting in a significant increase in the probability of traumatic fractures. Most patients with traumatic fractures have sudden onset and severe pain, so they often go to the emergency department for treatment.^21^

In the first aid of patients with fractures, medical staff often fix the fracture ends first to relieve muscle spasm, keep the internal dynamic balance of limbs, and promote swelling subsidence, thus laying the foundation for follow-up treatment.^22^ However, past clinical data have shown that medical staff will use splints for fixation in emergency treatment of patients with upper limb fractures. Despite the strong strength of the splint, it is too unlikely to change according to the shape of the fixation part so that a firm fixation cannot be achieved. Moreover, the poor air permeability of the splint may also cause secondary skin injury to patients after wearing it for a period of time.^23^

Adjustable upper limb fixator, as a new type of fractured upper limb fixator developed by medical staff in recent years, is mainly composed of basic splint wrapped with memory material and movable sliding plate material. It was developed to effectively control the circumference of fixator for the purpose of joint fixation. For patients with upper limb fractures, the adjustable upper limb fixator adopts targeted memory sponge at the part contacting the skin, and its polyurethane polymer material with open cell structure can protect the skin. Therefore, no corresponding complications such as skin damage will be triggered during the wearing period.^24^

It was also shown in this study that after treatment, the excellent and good rate (75.00%) in the observation group was higher than that in the control group (52.50%), the pain degree was better than that in the control group, and the total compliance rate (97.50%) was higher than that in the control group (75.00%). It shows that the upper limb fixator used in this study can promote fracture recovery to some extent. This is similar to the conclusion obtained by Nie XR^25^ in his research on the application of plastic splint in pre-hospital first aid for limb fracture. It is speculated that after the adjustable upper limb fixator is used for fixation, the fracture position is in an embedded state under the action of the contractile force between the muscles of the patient, and at the same time, it is in a state of transverse pressure due to the wrapping of the muscles on the bone, thereby maintaining the stability of the fracture end. This king of stability is dynamic, and long-term dynamic stability has a longitudinal squeezing effect on the fracture end, which can speed up the fracture healing.^26^-^28^

### Limitations of this study:

Fewer patients were included, and long-term efficacy observations were lacking. In future more patients will be included and long-term studies will be conducted to evaluate the value of fracture upper limb fixator in emergency treatment of patients with upper limb fractures.

## CONCLUSION

Fracture upper limb fixator shows various benefits in first aid of upper limb fractures, such as improving the clinical efficacy of patients with upper limb fractures, ameliorating pain, improving fitness effect, and reducing the probability of complications, which is of high clinical application value.

### Authors’ Contributions:

**LX and HS** carried out the studies, data collection, drafted the manuscript, are responsible and accountable for the accuracy and integrity of the work.

**JL** **and CZ** performed the statistical analysis and participated in its design.

**RY and** **MY** participated in acquisition, analysis, or interpretation of data and draft the manuscript. All authors read and approved the final manuscript.
